# Phylogenetic prediction of cis-acting elements: a *cre*-like sequence in Norovirus genome?

**DOI:** 10.1186/1756-0500-2-176

**Published:** 2009-09-07

**Authors:** Matías Victoria, Rodney Colina, Marize Pereira Miagostovich, José P Leite, Juan Cristina

**Affiliations:** 1 Laboratório de Virologia Comparada e Ambiental, Instituto Oswaldo Cruz, FIOCRUZ, Av. Brasil 4365, Manguinhos, 21040-360 Rio de Janeiro, RJ, Brasil; 2 Laboratorio de Virología Molecular. Centro de Investigaciones Nucleares, Facultad de Ciencias, Iguá 4225, 11400 Montevideo, Uruguay

## Abstract

**Background:**

Discrete RNA structures such as *cis*-acting replication elements (*cre*) in the coding region of RNA virus genomes create characteristic suppression of synonymous site variability (SSSV). Different phylogenetic methods have been developed to predict secondary structures in RNA viruses, for high-resolution thermodynamic scanning and for detecting SSSV. These approaches have been successfully in predicting cis-acting signals in different members of the family *Picornaviridae *and *Caliciviridae*. In order to gain insight into the identification of *cis*-acting signals in viruses whose mechanisms of replication are currently unknown, we performed a phylogenetic analysis of complete genome sequences from 49 Human Norovirus (NoV) strains.

**Findings:**

The complete coding sequences of NoV ORF1 were obtained from the DDBJ database and aligned. Shannon entropy calculations and RNAalifold consensus RNA structure prediction identified a discrete, conserved, invariant sequence region with a characteristic AAACG *cre *motif at positions 240 through 291 of the RNA dependant RNA polymerase (RdRp) sequence (relative to strain [EMBL:EU794713]). This sequence region has a high probability to conform a stem-loop.

**Conclusion:**

A new predicted stem-loop has been identified near the 5' end of the RdRp of Human NoV genome. This is the same location recently reported for *Hepatovirus cre *stem-loop.

## Findings

Internal base pairing that creates stem-loops and other RNA structures places constraints on sequence variability in bases required for structure formation in the genome of RNA viruses. For instance, the Hepatitis C virus (HCV) genome has a marked suppression of synonymous codon variability within several evolutionary conserved stem-loops in the core and NS5B coding regions that demonstrate their role in virus replication [1-3]. Discrete RNA structures such as cis-acting replication elements (*cre*) in the coding region of human enteroviruses (HEVs) [4] and other viruses also create characteristic suppression of synonymous site variability (SSSV), similar to that observed in HCV [5,6]. Different phylogenetic methods have been developed to predict secondary structures in RNA viruses, like PFOLD [2,7] or Alifold [8], for high-resolution thermodynamic scanning, and like UNAFold [9] for detecting SSSV [2]. These methods have permitted to identify suitable genome regions for an in-depth experimental analysis allowing establishing the role of the identified secondary RNA structures in translation or replication. This approach has permitted to raise the hypothesis that when SSSV (i.e. highly conserved synonymous sites in a RNA virus genome sequence alignment) takes place in a sequence region with a high probability of conforming a secondary structure (i.e. high probability of base pairing to generate a stable stem-loop), a cis-acting signal can be identified. This hypothesis has been successfully tested in different members of the family *Picornaviridae*, like Hepatitis A virus (HAV), Avian Encephalitis virus (AEV) and *Rhinovirus *[10,11] and in members of the family *Caliciviridae*, like *Norovirus*, *Sapovirus*, *Vesivirus *and *Lagovirus *[12].

In order to gain insight into the identification of cis-acting signals in viruses whose mechanisms of replication are currently unknown; we tested the above hypothesis for a group of 49 Human Noroviruses (NoV), for whom complete genome sequences have been recently obtained.

**Figure 1:**
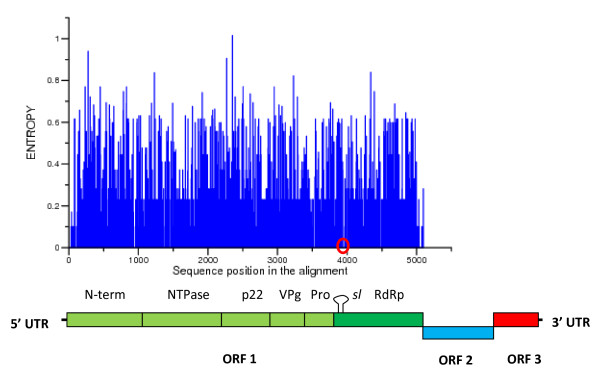
**Shannon entrophy in ORF1 of Human NoV strains**. The Shannon entropy at each position of the alignment is shown. Location of positions 3922 through 3973 of the alignment (which includes the AAACG motif) in a sequence region with zero entropy is shown by a red circle. A scheme showing the position of each gene in the NoV genome is shown below the graph. The location of the stem-loop is indicated *sl*.

The complete codes of ORF1 of 49 Human NoV were obtained from the DDBJ database and aligned using the MUSCLE program [13] (for strain names and accession numbers see Additional File 1). Once aligned, the Shannon entropy at each position of the sequence dataset was calculated [14]. This permitted to measure the relative variation in each site of a sequence alignment. The results of these studies are shown in Fig. 1.

Only few, discrete, genome regions in the ORF1 of Human NoV have a Shannon entropy of zero indicating that they are invariants among all NoV sequences introduced in this analysis (Fig. 1). Interestingly, one of these discrete regions has an AAACG *cre *sequence motif [10] at position 3948 to 3952 of the alignment. These positions correspond to positions 240 through 291 of the RNA dependant RNA polymerase (RdRp) sequence (relative to strain [EU794713]) (Fig. 1).

**Figure 2:**
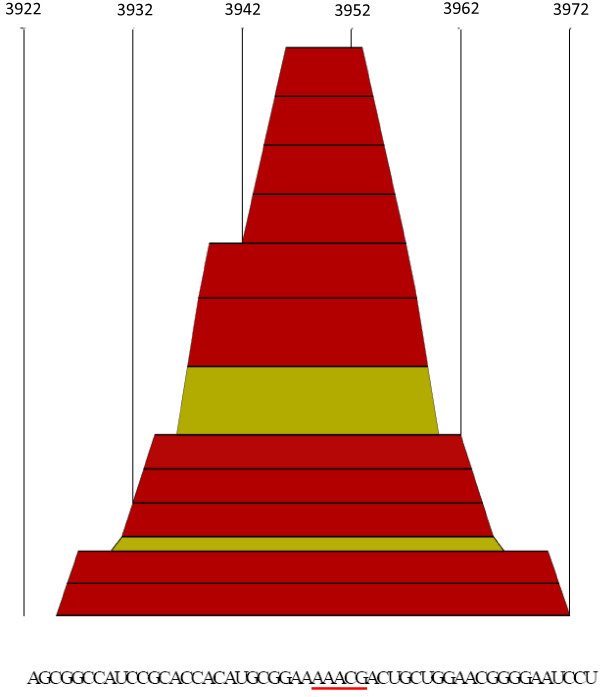
**"Mountain plot" of a NoV ORF1 sequence region with an entrophy equals to zero**. A mountain plot representing a secondary structure in a plot of height versus position is shown. Sequences from positions 3922 through 3973 of the alignment are shown at the bottom of the figure. Numbers at the top of the figure show site position in the alignment. Colors correspond to the Vienna RNA conservation coloring schema [16] (see also Fig. 3). Note that the AAACG motif (underlined in red) is predicted to be located in a loop of the secondary structure.

In order to observe if this *cre *motif is situated in a sequence region that has a high probability to conform a secondary structure [8,15], we used the RNAalifold Web Server [16]. First, we obtained a graphical representation of the secondary structure in a plot of height versus position, where the height m(*k*) is given by the number of base pairs enclosing the base at position *k *in the structure (i.e. loops correspond to plateaus, hairpin loops are peaks and helices to slopes). The results of these studies are shown in Fig. 2. The AAACG *cre *sequence motif is embedded in a sequence region with a very high probability to conform a stem-loop structure (see Fig. 2).

**Figure 3:**
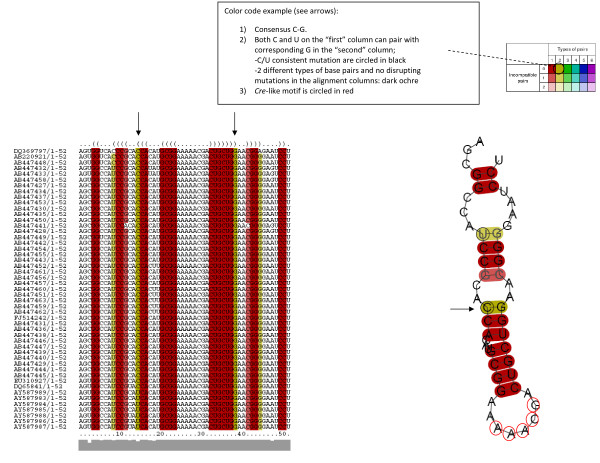
**Conservation of a predicted secondary structure in NoV ORF1 sequences**. Multiple sequence alignment across 49 Human NoV ORF1 sequences shows a consensus secondary structure from positions 3922 through 3973 of the NoV genome (positions 240 through 291 of the RdRp sequence, relative to strain [EMBL:EU794713]). The structure is shown in the dot-bracket format [16] above the alignment. Each corresponding bracket represents consensus base pairs of the alignment columns beneath. Sequences are color-coded according to consistent and compensatory mutations in the aligned sequences regarding the conserved structure (see figure text box). Pale colors indicate that a base-pair cannot be formed in some sequences of the alignment. The sequence conservation profile is shown in gray bars bellow the alignment. The conserved predicted secondary structure, color-coded according to the different types of base pairs in the corresponding alignment columns, is shown on the right side of the figure.

Then, the consensus RNA structures of the selected alignment region were folded using the RNAalifold program [16]. The resulting structures as well as the alignments were color-coded according to a coloring scheme for highlighting the mutational pattern with respect to the structure (Vienna RNA conservation coloring schema) [16]. The results of these studies are shown in Fig. 3.

Using the RNAalifold program [16] we have identified the presence of a unique and conserved stem-loop near the 5' end of the RdRp coding region for the 49 Human NoV genomes analyzed (Figs. 1 and 3). This predicted structure contains a *cre *sequence motif (Fig. 3). Interestingly, the stem-loop predicted for Human NoV is situated near the 5' end of the RdRp (Figs. 2 and 3). This is the same location recently reported for *Hepatovirus cre *stem-loop [10] (Fig. 1).

RNA structure predictions are consistent with previous analyses based on the thermodynamic folding of individual sequences [12,17,18]. Although RNA structure is clearly not the only cause of SSSV, occurring for example also in overlapping gene sequences [19], there is an impressive co-localization of the major sites of SSSV and thermodynamically predicted secondary structures [4,11,12].

NoV belong to the family *Caliciviridae*, and they are non-enveloped viruses with positive, single-stranded RNA genomes. They also share other important features with picornaviruses, like having a VPg protein covalently linked to the 5' of the genomic RNA [20]. Nevertheless, in contrast with picornaviruses, NoV express a downstream sub-genomic (sg) transcript encoding structural genes [20]. NoV are the leading cause of outbreaks of acute gastroenteritis in humans worldwide [21].

Despite the importance of these outbreaks, our understanding of the RNA structures or sequences required for NoV replication has been limited. Previous reports have identified the poly-pyrimidine tract-binding protein (PTB), poly-A binding protein (PAB) and La autoantigen to interact with the 3' untranslated region of the Norwalk virus genome [17]. Very recent studies have identified *cis*-acting signals in the 5' and 3' regions as well as at the start of the sg RNA transcript of NoV [12].

As a member of the family *Caliciviridae*, NoV are thought to replicate in a manner typical of positive-stranded RNA viruses, through the synthesis of a full-length anti-genomic strand (reverse complement copy) using the viral RdRp translated initially from the RNA genome entering the cell [22]. The minus strand then acts as a template for the synthesis of full-length genomic RNA from which non-structural proteins are translated, including the RdRp. Features of the RdRp common to all positive-sense RNA viruses support this idea [23].

Although the presence of this new putative cis-acting signal predicted in this study was not yet investigated *in vitro *due to the lack of a standard cell culture to grow these viruses, the probability that this predicted structure will acts as a functional element may open new avenues to our understanding of molecular mechanisms of NoV replication.

Extensive mutagenesis studies performed in members of the family *Picornaviridae*, like Poliovirus (PV) and Human Rhinovirus 14 (HRV-14), revealed a critical conserved AAACA/G *cre *sequence motif in the 5' half of the loop sequence that is essential for its function [10]. Similar conserved motifs are present within the loops of the *cre *elements of other picornaviruses and are important for RNA replication [10,24,25].

Paul and colleagues (2003) have shown that the PV *cre *act as the template for VPg uridylylation through a "slide-back" mechanism catalyzed by the 3D^pol ^(RdRp) [24,26,27]. The uridylylation of VPg leads to the production of VPg-pUpU, which serves as the protein primer for new RNA synthesis [28].

Interestingly, recent studies have shown that incubation of VPg with NoV 3D^pol ^(RdRp) generates VPg-poly(U) and that this uridylylated VPg can prime the replication of polyadenylated RNA [29]. In contrast, replication of antigenomic RNA was not primer dependent. Moreover, on nonpolyadenylated RNA, NoV RdRp initiated RNA synthesis *de novo *[29]. These findings clearly show that initiation of replication of the NoV genome by the RpRp requires a VPg-protein-primed initiation of replication of polyadenylated genomic RNA and a *de novo *initiation of replication of antigenomic RNA [29]. Besides, very recent studies revealed that the NoV RdRp is a typical template-dependent RNA polymerase [30].

It is possible that the predicted stem-loop identified near the 5' end of the NoV RdRp coding region, which share a *cre*-like sequence motif with members of the family *Picornaviridae *[10], will be capable to perform the uridylylation of VPg. If that is the case, this will permit VPg to act as a primer for the synthesis of the minus strand RNA, in agreement with the results outlined above [30].

## Competing interests

The authors declare that they have no competing interests.

## Authors' contributions

JC conceived the study. MV and JC designed, performed and revised the phylogenetic analysis. MV, RC, MPM and JPL contributed to the interpretation and discussion of the results found. JC wrote the manuscript. All authors read and approved the final manuscript.

## Supplementary Material

Additional file 1 **Origins of Norovirus strains**. The table provides the strain names and accession numbers of their respective sequences of ORF1 of Norovirus strains included in this study. Click here for file
