# Supplementary material for: Phylogenetic prediction of cis-acting elements: a cre-like sequence in Norovirus genome?
Source: BMC Res Notes. 2009 Sep 7;2:176. doi: 10.1186/1756-0500-2-176 (PMC2749865; doi:10.1186/1756-0500-2-176)
Supplement: Additional file 1 — Origins of Norovirus strains. The table provides the strain names and accession numbers of their respective sequences of ORF1 of Norovirus strains included in this study. [file 1756-0500-2-176-S1.doc]

| **Additional File 1. Origins of Norovirus strains.** | |
| --- | --- |
| **Name** | **Accession Number** |
| Hu/Guangzhou/NVgz01/CHN | [EMBL:DQ369797] |
| Hu/Chiba/04-1050/2005/JP | [EMBL:AB220921] |
| Hu/GII-4/Sakai2/2006/JP | [EMBL:AB447448] |
| Hu/GII-4/Aomori1/2006/JP | [EMBL:AB447432] |
| Hu/GII-4/Aomori2/2006/JP | [EMBL:AB447433] |
| Hu/GII-4/Saga5/2006/JP | [EMBL:AB447458] |
| Hu/GII-4/Hokkaido1/2006/JP | [EMBL:AB447427] |
| Hu/GII-4/Aomori4/2006/JP | [EMBL:AB447434] |
| Hu/GII-4/Akita2/2006/JP | [EMBL:AB447453] |
| Hu/GII-4/Hokkaido4/2006/JP | [EMBL:AB447430] |
| Hu/GII-4/Aomori5/2006/JP | [EMBL:AB447450] |
| Hu/GII-4/Miyagi4/2006/JP | [EMBL:AB447441] |
| Hu/GII-4/Aomori5/2006/JP | [EMBL:AB447435] |
| Hu/GII-4/Akita2/2006/JP | [EMBL:AB447437] |
| Hu/GII-4/Miyagi4/2006/JP | [EMBL:AB447428] |
| Hu/GII-4/Sakai3/2006/JP | [EMBL:AB447449] |
| Hu/GII-4/Miyagi5/2006/JP | [EMBL:AB447442] |
| Hu/GII-4/Ehime2/2006/JP | [EMBL:AB447454] |
| Hu/GII-4/Ehime5/2006/JP | [EMBL:AB447455] |
| Hu/GII-4/Toyama1/2006/JP | [EMBL:AB447443] |
| Hu/GII-4/Hiroshima2/2006/JP | [EMBL:AB447452] |
| Hu/GII-4/Kumamoto3/2006/JP | [EMBL:AB447461] |
| Hu/GII-4/Saga1/2006/JP | [EMBL:AB447456] |
| Hu/GII-4/Saga4/2006/JP | [EMBL:AB447457] |
| Hu/GII-4/Kumamoto2/2006/JP | [EMBL:AB447460] |
| Hu/GII-4/Hiroshima1/2006/JP | [EMBL:AB447451] |
| Hu/GII-4/Kumamoto5/2006/JP | [EMBL:AB447463] |
| Hu/GII-4/Kumamoto1/2006/JP | [EMBL:AB447459] |
| Hu/GII-4/Kumamoto4/2006/JP | [EMBL:AB447462] |
| Hu/GII-4/CUK-3/2008/KR | [EMBL:FJ514242] |
| Hu/GII-4/Hokkaido5/2006/JP | [EMBL:AB747431] |
| Hu/GII-4/Akita1/2006/JP | [EMBL:AB447436] |
| Hu/GII-4/Akita4/2006/JP | [EMBL:AB447438] |
| Hu/GII-4/Aichi3/2006/JP | [EMBL:AB447446] |
| Hu/GII-4/Aichi4/2006/JP | [EMBL:AB447447] |
| Hu/GII-4/Akita5/2006/JP | [EMBL:AB447439] |
| Hu/GII-4/Miyagi2/2006/JP | [EMBL.AB447440] |
| Hu/GII-4/Hokkaido3/2006/JP | [EMBL:AB447429] |
| Hu/GII-4/Toyama4/2006/JP | [EMBL:AB447444] |
| Hu/GII-4/Toyama5/2006/JP | [EMBL:AB447445] |
| Hu/Houston/TCH186/2002/US | [EMBL:EU310927] |
| Hu/GII-4/MD-2004/2004/US | [EMBL:DQ658413] |
| Hu/GII-4/MD-2004/2004/US | [EMBL:AY587989] |
| Hu/NLV/Oxford/B4S2/2002/UK | [EMBL:AY587983] |
| Hu/NLV/Oxford/B4S5/2002/UK | [EMBL:AY587984] |
| Hu/NLV/Oxford/B4S6/2002/UK | [EMBL:AY587985] |
| Hu/NLV/Oxford/B4S1/2002/UK | [EMBL:AY587988] |
| Hu/NLV/Oxford/B4S4/2002/UK | [EMBL:AY587986] |
| Hu/NLV/Oxford/B4S7/2002/UK | [EMBL:AY587987] |
